# Supplementary material for: The remanufacturing evaluation for feasibility and comprehensive benefit of retired grinding machine
Source: PLoS One. 2020 Jun 18;15(6):e0234603. doi: 10.1371/journal.pone.0234603 (PMC7302915; doi:10.1371/journal.pone.0234603)
Supplement: Attachment — Submitted filename: PONE-D-19-28523_reviewer.pdf [file pone.0234603.s002.pdf]

PLOS ONE

The Remanufacturing Evaluation for Feasibility and Comprehensive Benefit of Retired Grinding Machine

--Manuscript Draft--

**Manuscript Number:**	PONE-D-19-28523
**Article Type:**	Research Article
**Full Title:**	The Remanufacturing Evaluation for Feasibility and Comprehensive Benefit of Retired Grinding Machine
**Short Title:**	The Remanufacturing Evaluation for Feasibility and Comprehensive Benefit of Retired Grinding Machine
**Corresponding Author:**	Tianbai Ling<br>Shanghai University<br>shanghai, shanghai CHINA
**Keywords:**	remanufacturing; retired grinder; feasibility evaluation; comprehensive benefit evaluation
**Abstract:**	As the final form of recycling, remanufacturing can restore the old products with high added value to a new state. At the same time, it achieves enormous energy-saving and emission reduction benefits. Grinding machine is widely used in aerospace, military, automobile and precision processing industries. It is the key equipment of manufacturing industry. Grinding process is to obtain the final dimension accuracy and surface finish of core parts. It is usually the last step in the manufacturing process of parts. Therefore, grinding machine is a typical high value-added mechanical and electrical products, which can be remanufactured and reused, and its life cycle is extended. This paper establishes a feasibility evaluation model for grinder remanufacturing, and evaluates the remanufacturing capability of retired grinders from the aspects of technology, economy, resources and environment. A comprehensive benefit evaluation model of remanufacturing grinder was established by combining Analytic Hierarchy Process (AHP) with Expert Investigation Method (Delphi). The analysis results of grinder remanufacturing case shows that the mechanical performance and processing accuracy of the remanufactured grinder can meet the standard of the new machine tool, and some indexes are even better. Case analysis proves that grinder remanufacturing has good residual value and comprehensive benefits, and provides practical experience and data for the remanufacturing process of retired grinders.
**Order of Authors:**	Tianbai Ling
	Yongyi He
**Additional Information:**	
**Question**	**Response**
**Financial Disclosure**<br><br>Enter a financial disclosure statement that describes the sources of funding for the work included in this submission. Review the submission guidelines for detailed requirements. View published research articles from *PLOS ONE* for specific examples.<br><br>This statement is required for submission and **will appear in the published article** if the submission is accepted. Please make sure it is accurate.	Ling Tianbai (LTB)<br>Z16030384<br>Shanghai Science and Technology Development Foundation (CN)<br>http://www.stefg.org/mobile/about/inventory.aspx?key=&hangye=&fenhui=72634837642510336<br>no sponsors or funders play any role in the study design, data collection and analysis, decision to publish, or preparation of the manuscript.

Powered by Editorial Manager® and ProduXion Manager® from Aries Systems Corporation

**Unfunded studies**<br>Enter: *The author(s) received no specific funding for this work.*<br><br>**Funded studies**<br>Enter a statement with the following details:<br>• Initials of the authors who received each award<br>• Grant numbers awarded to each author<br>• The full name of each funder<br>• URL of each funder website<br>• Did the sponsors or funders play any role in the study design, data collection and analysis, decision to publish, or preparation of the manuscript?<br>• **NO** - Include this sentence at the end of your statement: *The funders had no role in study design, data collection and analysis, decision to publish, or preparation of the manuscript.*<br>• **YES** - Specify the role(s) played.<br><br>* typeset	
**Competing Interests**<br><br>Use the instructions below to enter a competing interest statement for this submission. On behalf of all authors, disclose any competing interests that could be perceived to bias this work—acknowledging all financial support and any other relevant financial or non-financial competing interests.<br><br>This statement **will appear in the published article** if the submission is accepted. Please make sure it is accurate. View published research articles from *PLOS ONE* for specific examples.	The authors have declared that no competing interests exist.

Powered by Editorial Manager® and ProduXion Manager® from Aries Systems Corporation

**NO authors have competing interests**<br><br>Enter: *The authors have declared that no competing interests exist.*<br><br>**Authors with competing interests**<br><br>Enter competing interest details beginning with this statement:<br><br>*I have read the journal's policy and the authors of this manuscript have the following competing interests: [insert competing interests here]*<br><br>* typeset	
**Ethics Statement**<br><br>Enter an ethics statement for this submission. This statement is required if the study involved:<br><br>• Human participants<br>• Human specimens or tissue<br>• Vertebrate animals or cephalopods<br>• Vertebrate embryos or tissues<br>• Field research<br><br>Write "N/A" if the submission does not require an ethics statement.<br><br>General guidance is provided below. Consult the submission guidelines for detailed instructions. **Make sure that all information entered here is included in the Methods section of the manuscript.**	the submission does not require an ethics statement.

**Format for specific study types**<br><br>**Human Subject Research (involving human participants and/or tissue)**<br>• Give the name of the institutional review board or ethics committee that approved the study<br>• Include the approval number and/or a statement indicating approval of this research<br>• Indicate the form of consent obtained (written/oral) or the reason that consent was not obtained (e.g. the data were analyzed anonymously)<br><br>**Animal Research (involving vertebrate animals, embryos or tissues)**<br>• Provide the name of the Institutional Animal Care and Use Committee (IACUC) or other relevant ethics board that reviewed the study protocol, and indicate whether they approved this research or granted a formal waiver of ethical approval<br>• Include an approval number if one was obtained<br>• If the study involved *non-human primates*, add *additional details* about animal welfare and steps taken to ameliorate suffering<br>• If anesthesia, euthanasia, or any kind of animal sacrifice is part of the study, include briefly which substances and/or methods were applied<br><br>**Field Research**<br><br>Include the following details if this study involves the collection of plant, animal, or other materials from a natural setting:<br>• Field permit number<br>• Name of the institution or relevant body that granted permission	
**Data Availability**<br><br>Authors are required to make all data underlying the findings described fully available, without restriction, and from the time of publication. PLOS allows rare exceptions to address legal and ethical concerns. See the PLOS Data Policy and FAQ for detailed information.	Yes - all data are fully available without restriction

A Data Availability Statement describing where the data can be found is required at submission. Your answers to this question constitute the Data Availability Statement and **will be published in the article**, if accepted.<br><br>**Important:** Stating 'data available on request from the author' is not sufficient. If your data are only available upon request, select 'No' for the first question and explain your exceptional situation in the text box.<br><br>Do the authors confirm that all data underlying the findings described in their manuscript are fully available without restriction?	
**Describe where the data may be found in full sentences. If you are copying our sample text, replace any instances of XXX with the appropriate details.**<br><br>• If the data are **held or will be held in a public repository**, include URLs, accession numbers or DOIs. If this information will only be available after acceptance, indicate this by ticking the box below. For example: *All XXX files are available from the XXX database (accession number(s) XXX, XXX.*).<br>• If the data are all contained **within the manuscript and/or Supporting Information files**, enter the following: *All relevant data are within the manuscript and its Supporting Information files.*<br>• If neither of these applies but you are able to provide **details of access elsewhere**, with or without limitations, please do so. For example:<br><br>*Data cannot be shared publicly because of [XXX]. Data are available from the XXX Institutional Data Access / Ethics Committee (contact via XXX) for researchers who meet the criteria for access to confidential data.*<br><br>*The data underlying the results presented in the study are available from (include the name of the third party*	All relevant data are within the manuscript and its Supporting Information files.

and contact information or URL).<br>• This text is appropriate if the data are owned by a third party and authors do not have permission to share the data.<br><br>* typeset	
Additional data availability information:	Tick here if the URLs/accession numbers/DOIs will be available only after acceptance of the manuscript for publication so that we can ensure their inclusion before publication.; Tick here if your circumstances are not covered by the questions above and you need the journal's help to make your data available.

Manuscript
Click here to download Manuscript The Remanufacturing Evaluation for Feasibility (1).pdf

The Remanufacturing Evaluation for Feasibility and Comprehensive Benefit of Retired Grinding Machine

Tianbai Ling and Yongyi He
School of Mechatronic Engineering and Automation, Shanghai University, Shanghai 200072, China
Correspondence should be addressed to Tianbai Ling; lingskywhite@shu.edu.cn

Abstract: As the final form of recycling, remanufacturing can restore the old products with high added value to a new state. At the same time, it achieves enormous energy-saving and emission reduction benefits. Grinding machine is widely used in aerospace, military, automobile and precision processing industries. It is the key equipment of manufacturing industry. Grinding process is to obtain the final dimension accuracy and surface finish of core parts. It is usually the last step in the manufacturing process of parts. Therefore, grinding machine is a typical high value-added mechanical and electrical products, which can be remanufactured and reused, and its life cycle is extended. This paper establishes a feasibility evaluation model for grinder remanufacturing, and evaluates the remanufacturing capability of retired grinders from the aspects of technology, economy, resources and environment. A comprehensive benefit evaluation model of remanufacturing grinder was established by combining Analytic Hierarchy Process (AHP) with Expert Investigation Method (Delphi). The analysis results of grinder remanufacturing case shows that the mechanical performance and processing accuracy of the remanufactured grinder can meet the standard of the new machine tool, and some indexes are even better. Case analysis proves that grinder remanufacturing has good residual value and comprehensive benefits, and provides practical experience and data for the remanufacturing process of retired grinders.

Keywords: remanufacturing; retired grinder; feasibility evaluation; comprehensive benefit evaluation.

List of symbols

T Criteria of technology feasibility
t_d Ease of disassembly
t_c Feasibility of cleaning
t_i Feasibility of inspection and sorting
t_r Feasibility of part reconditioning
α Wear condition of guide rail
C Criteria of economic feasibility
C_1 Grinding machine remanufacturing cost
C_2 Manufacturing cost of new grinding machine
f The ratio of C_1 to C_2
E Criteria of resources environment feasibility
R Resource utilization rate
P Energy conservation rate
η Environmental protection rate

1. Introduction

1.1. Development of remanufacturing abroad

From 1930s to 1940s, American automobile manufacturing industry took the lead in remanufacturing practice. After nearly 80 years of development, the developed countries in Europe and America, represented by the United States, Germany, the United Kingdom, the Netherlands and Japan, have established a remanufacturing industry system covering many units, such as waste product recycling and disassembly detection, remanufacturing production control, remanufacturing technology research development and remanufacturing product marketing [1,2]. The remanufactured products cover many fields, such as aerospace, heavy off-road equipment, automotive parts, locomotives, information technology products, electrical equipment, medical equipment and office appliances. It have formed perfect remanufacturing operation mode and mature remanufacturing market environment. The annual output value of the U.S. remanufacturing industry rose rapidly from $75 billion in 2005 to $120 billion in 2017, The United States has become the largest country in the world in remanufacturing industry, and the annual output value of the global remanufacturing

industry rose from $100 billion in 2005 to $170 billion in 2017. Therefore, the remanufacturing industry is called "giant in industry" in foreign countries.

Machine tool is a kind of power mechanical device with complex structure, which manufactures mechanical parts by metal cutting. Grinding machine, as a typical mechanical and electrical equipment, has a high value of recycling and remanufacturing. The waste grinder can be recycled and utilized as well as the iron and steel resources, thus greatly saving iron and steel resources. The cost of remanufacturing grinder can be reduced by more than 40% compared with the purchase of new machine tools, the durability of the mechanical part of grinder is strong and stable, especially for the bed and column castings, the longer the aging, the better the performance and reliability, and the grinder is suitable for recycling and remanufacturing. The product and functional components have good interchangeability, which greatly simplifies the remanufacturing process.

Remanufacturing, as a specific type of recycling, makes the fact that the used durable goods can be repaired to a condition like new realized. By means of remanufacturing, most of the used machinery parts can be repaired to a condition like new with warranty to match, which not only alleviates environmental contamination, but reduces energy consumption and professional labor used in production.the most importance is that the product performance after remanufacturing should reach or exceed the original product [3-5].

Throughout the development of remanufacturing industry abroad, the United States, Europe, Japan and other developed countries and regions of remanufacturing industry have done a lot of work in the aspects of remanufacturing technology management and practice of remanufacturing enterprises. The machine tool remanufacturing in the United States has experienced the development stages of maintenance, renovation, Numerical Control (NC) transformation and remanufacturing. After years of development and with the elimination of a large number of enterprises in the market, there are more than 300 enterprises specializing in machine tool remanufacturing in the United States, mainly third-party machine tool remanufacturing service providers [6,7]. Maintenance Service Corp. has a 60-year history of machine tool modification and remanufacturing. They can transform, renovate and remanufacture all kinds of machine tools. The company has completed more than 20,000 machines in total. Machine Tool Builders, Inc. has been engaged in machine tool remanufacturing business for nearly 20 years. They mainly remanufactures gear processing machine tools. At present, The company has the ability to design and manufacture new machine tools, and has completed a number of machine tool remanufacturing business. In Japan, the manufacturing industry breaks away from the traditional maintenance concept of old machine tools and equipment in order to modernize them by remanufacturing. According to statistics, there are at least 20 enterprises engaged in machine tool remanufacturing and have a certain scale in Japan, such as Osaka Engineering Company, Nozaki Engineering Company, Yamazaki Mazak Company, etc.

1.2. Development of remanufacturing in China

Machine tool remanufacturing industry has developed rapidly in recent years in China. The promotion by centralized environmental legislation, environmental awareness of customers has been continuously enhanced. the economic and social benefits of remanufacturing enterprises and customers have been continuously improved. However, due to the lack of key remanufacturing technology, maintenance and modification are still the main services of machine tool remanufacturing, which can not guarantee its performance and reliability.

Since 1999, government has vigorously publicized and deeply studied remanufacturing project in China. In 2005, Jinan Fuqiang Power Co., Ltd. was recognized as a pilot unit of national circular economy, which indicates China's willingness to make strategic attempts in remanufacturing; In 2010, eleven ministries and commissions jointly issued that Opinions on Promoting the Development of Remanufacturing Industry, which is an important milestone of remanufacturing in China; In order to implement the Circular Economy Promotion Law in 2013, Chinese government strongly supports the promotion and use of remanufactured products, promotes the recycling of used remanufactured parts and enlarges the market share of remanufactured products; In 2015, the government has issued the Outline of Made-in-China 2025 and proposed to implement high-end remanufacturing and intelligent remanufacturing to promote the sustainable development of remanufacturing industry [8].

Machine tool remanufacturing enterprises mainly include original machine tool manufacturing enterprises and independent third-party machine tool remanufacturing enterprises in China. Chongqing Machine Tool Group Co., Ltd. regards machine tool remanufacturing as one of the major strategies of the enterprise, and gradually make the remanufacturing of ordinary mechanical gear hobbing machine a new growth point of enterprise profits. In addition, Shenyang Machine Tool Group, Dalian Machine Tool Group, Shanghai Machine Tool Works Co., Ltd and other companies are engaged in machine tool remanufacturing services.

Along with the rapid increase in living standard, the consumption of energy and non-renewable material is rapidly reaching, what many experts believe, unsustainable levels, which poses significant environmental challenges [9]. Remanufacturing is a powerful product recovery option which generates products as good as new ones from old discarded ones. This technique can also help to reduce the environmental impact of the product in its final disposal. Growing concern for resource conservation and waste reduction led to the augmentation of remanufacturing. This paper presents a feasibility evaluation method for grinder remanufacturing, which evaluates the technical feasibility, economic feasibility and resource environment feasibility, provides information support for grinder remanufacturing [10-13].

2. Analysis of grinding machine remanufacturing process

2.1. Introduction of grinding machine structure

The main features of high-speed Computer Numerical Control (CNC) grinder include high static stiffness and automation of the whole grinder, high processing accuracy, reliability and stability, and convenient use and maintenance. Workbench is driven by ball screw pair, the minimum setting unit is 0.1um. The grinding wheel spindle system adopts the hydrodynamic spindle system, which has the characteristics of high stiffness, high rotation accuracy, high grinding efficiency, less vibration and strong bearing capacity under working conditions. CNC grinder adopts fully enclosed external protective device, which has a high safety factor. At the same time, the internal electrical machinery chain is stable and reliable to ensure safe production.

CNC grinding machine is T-shaped overall layout, in which as an independent module, there are electric box, cooling and filtering system, hydraulic station and CNC panel control box. The overall structure of grinder consists of bed, motor, worktable, headframe, tailstock and grinding wheel spindle system. The overall three-dimensional model is shown in Fig. 1.

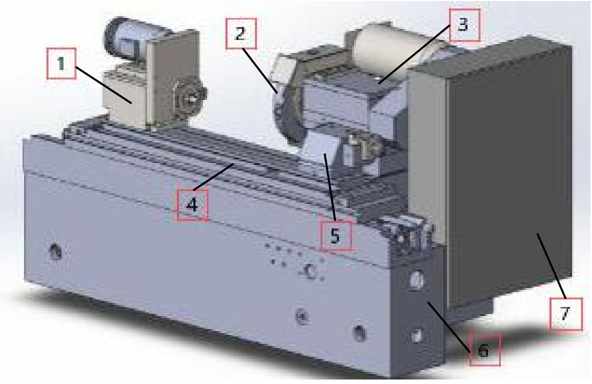

Figure 1: The overall three-dimensional model of grinding machine.
1: headframe, 2: grinding wheel, 3: wheelhead frame spindle system,
4: workbench, 5: tailstock, 6: bed, 7: electrical control box.

Workbench is installed on the guide rail of the front bed of the grinder. Headframe and tailstock are installed at both ends of the workbench. According to the different processing requirements and the shape of the parts, Mohs taper top, chuck or pneumatic chuck can be installed between the headframe and tailstock. Due to the different processing lengths of different parts, the tailstock adopts linear guide rail, which has high guiding accuracy and can be moved longitudinally for clamping operation.

The grinding wheel spindle system is installed on the guide rail of the back bed of the grinder, and the spindle system is the most critical core component of the grinder. The spindle system can move on the transverse guide rail to realize the axial feeding motion and to adjust the processing distance between the grinding wheel and the workpiece conveniently. The grinder spindle is supported by hydrodynamic and hydrostatic bearings, the spindle system is equipped with an independent lubrication device, which improves the life of bearings and spindles.

Because the grinder structure needs to meet the requirements of high processing accuracy, high rigidity and strong bearing capacity, the grinder guideways are basically in the form of integrated guideways that is hard rails. As shown in Fig. 2, the guide rails and the bed are integrated casting parts, which are processed on the basis of the bed castings, and then processed into guideways by quenching and grinding. Machine tools such as lathes, milling machines and machining centers are basically in the form of linear guideways. As shown in Figure 3, linear guides and machine bed

are separated structures. After the guides are worn out, they can be disassembled and replaced. The structure is simple and the processing is convenient, but the rigidity and bearing capacity of linear guides are worse than that of hard ones.

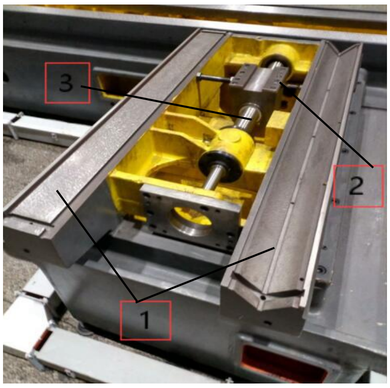

Figure 2: Integrated guideways.
1: integrated bed guideways, 2: nut seat, 3: ball screw.

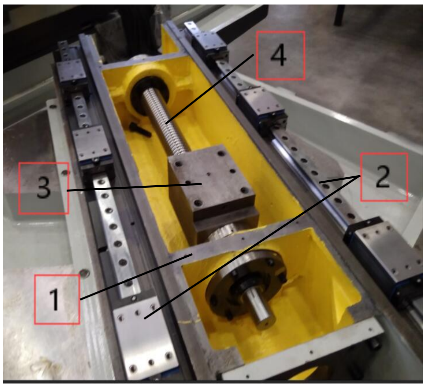

Figure 3: Linear guideways.
1: bed, 2: linear guideways, 3: nut seat, 4: ball screw.

2.2. Grinding machine remanufacturing process

The model of grinder remanufacturing can be divided into contract remanufacturing and commercial remanufacturing. Because there are many uncertainties in the quality grading process of recycled waste grinders, the remanufacturing process is more complex than that of new machine tools. Therefore, in the remanufacturing market of China, the remanufacturing grinders is mainly based on contract remanufacturing mode, which provides corresponding remanufacturing machine tools according to customer needs [14,15].

Due to the limitation of existing structure and material, the remanufacturing process of waste grinder must be adapted to the current situation of products. As shown in Fig. 4, grinder remanufacturing is a systematic organization. Firstly according to the flow chart of remanufacturing production of waste grinder, it is determined that the machine tool can meet the remanufacturing standard before it can be recycled. Secondly the process steps of disassembly, cleaning, inspection and classification are implemented for the parts of waste machine tool that meet the remanufacturing evaluation standard, and the remanufactured parts of waste machine tool are stored in the storage area to be processed. Finally the surface treatment technology is used to re-process the remanufactured parts. The remanufactured parts and the purchased new parts are reassembled and the remanufactured grinder is generated. The scrap grinder parts after disassembly, cleaning and testing can be divided into four categories according to their damage status: direct reusable parts, remanufactured parts, recyclable parts and direct scrap parts [16,17].

Through the remanufacturing of grinders, the residual value of waste grinders can be fully reused. The performance of remanufacturing grinders can be equal to or better than that of new machine tools, and the functions that new machine tools do not have can be increased, but the cost is only 40% to 70% of new machine tools.

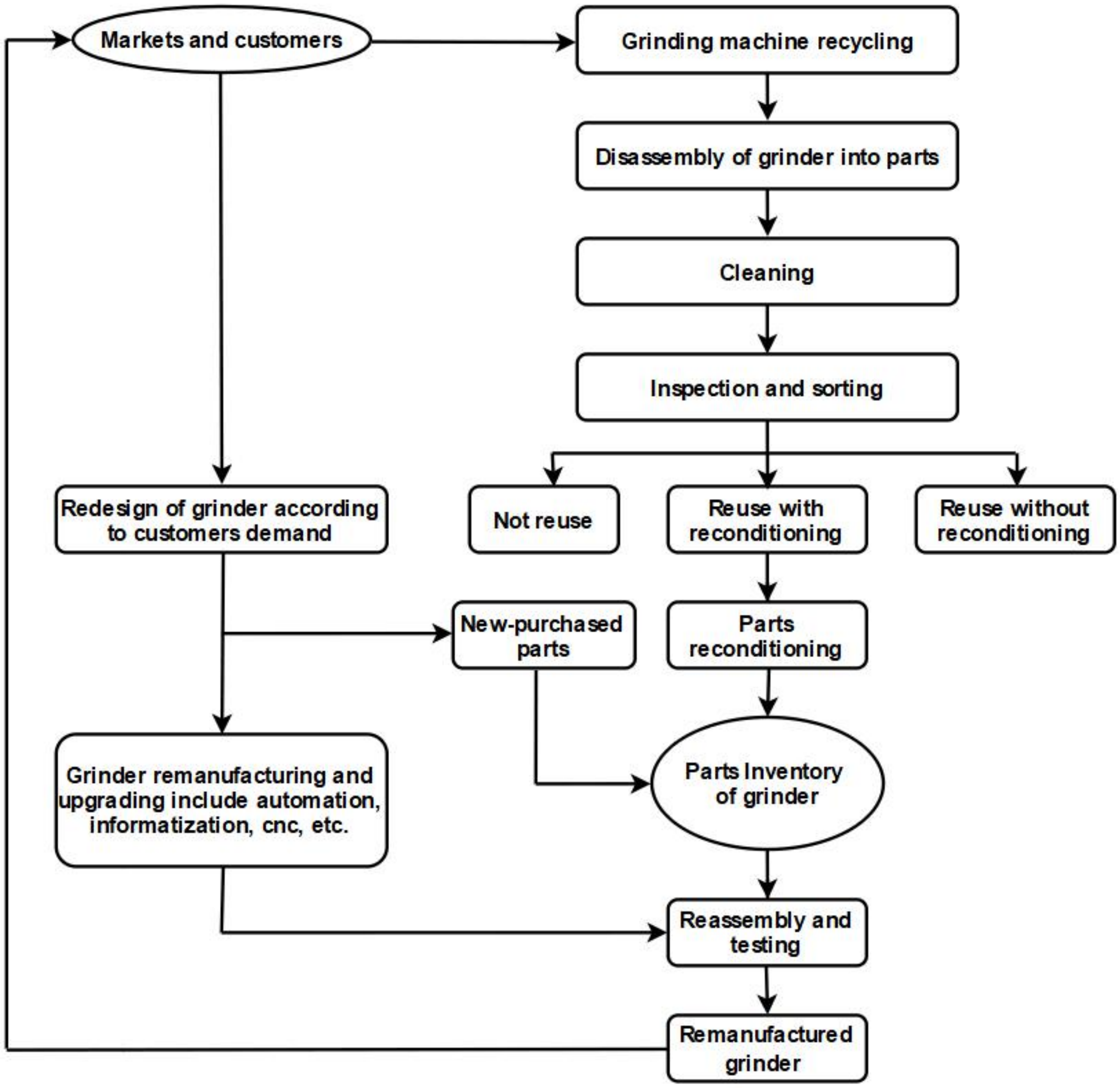

Figure 4: Process system of grinding machine remanufacturing.

3. Feasibility evaluation for the remanufacturability of waste grinding machine

3.1. Evaluating process of grinding machine remanufacturability

Based on the analysis of grinder remanufacturing process model, this paper establishes a comprehensive evaluation method model of the remanufacturing feasibility of waste grinders based on expert experience knowledge, as shown in Fig. 5. Firstly, considering the serious damage and wear of the recycled waste grinder, the remanufacturing of the grinder may be difficult to implement, so it is necessary to analyze the technical feasibility of the remanufacturing of the waste grinder to ensure that the machine can use modern manufacturing, information, numerical control and automation technology to achieve its functional recovery and performance improvement. If the technical feasibility of remanufacturing of the grinder is poor, then only the material recovery and recycling treatment can be carried out. Secondly, the cost of remanufacturing of the waste grinder with better technical feasibility needs to be calculated and evaluated. It is necessary to determine whether the remanufacturing of the grinder can make the remanufacturer profitable. If not, the remanufacturer will adopt material recovery and other methods to deal with the remanufacturing cost. The third step is to analyze the resource and environmental benefits of grinder remanufacturing from the perspectives of material saving, energy saving and environmental emission reduction. The purpose of remanufacturing is to save resources and reduce emissions by recycling and reusing waste resources. Finally, the waste grinder with better remanufacturing feasibility enters disassembly, cleaning, testing and classification. In the process of repairing, reprocessing and reassembling, the performance level of new machine tools can be achieved by means of precision recovery, function recovery and performance improvement, while the machine tools with poor feasibility of remanufacturing can be recycled by means of material recovery.

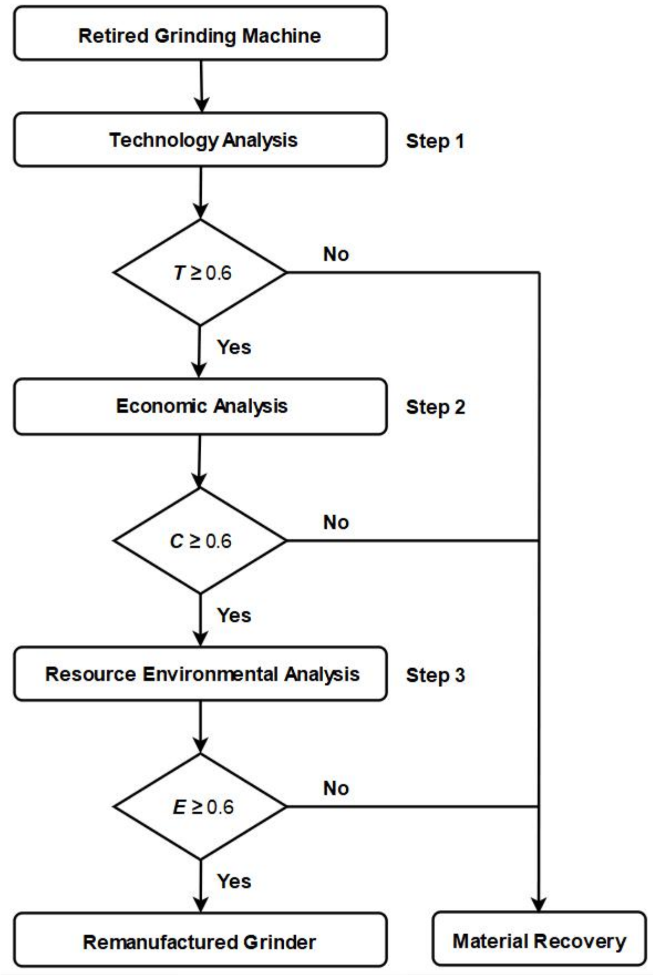

Figure 5: Evaluating process of grinding machine remanufacturability.

On the basis of data investigation and case analysis of grinder remanufacturing industry, it is concluded that the feasibility evaluation of waste grinder remanufacturing is mainly considered from three aspects which is technical feasibility, economic feasibility, resource and environment feasibility. The feasibility evaluation system of grinder remanufacturing is shown in Table 1. The evaluation criteria are mainly composed of three parts, that is technical feasibility, economic feasibility and resource and environment feasibility. Each criterion can be subdivided into various indicators.

Table 1: Feasibility evaluation system of grinder remanufacturing

Criteria	Index	Notions
Technology feasibility	Ease of disassembly	t_d
	Feasibility of cleaning	t_c
	Feasibility of inspection and sorting	t_i
	Feasibility of part reconditioning	t_r
Economic feasibility	Comprehensive economic feasibility	C
Resources environment feasibility	Material and energy saving	E

3.2. Criteria of technology feasibility

The technological feasibility of remanufacturing of waste grinders should be considered from all stages of remanufacturing process, including the condition of the bed guide of casting, the simplicity of disassembly, the feasibility of cleaning, the feasibility of detection and classification, and the feasibility of parts and components

remanufacturing. Disassembly is the first step in the remanufacturing process and the basis and premise for the scrap grinder to enter the remanufacturing process. Because remanufacturing is not considered in the original design process of the waste grinder, parts of the waste grinder may be damaged in the disassembly process.

The disassembly of grinder remanufacturing is different from material recovery and recycling [18]. The damage of parts caused by disassembly should be minimized and the disassembly efficiency should be improved. There are various kinds of bolts, pins and other connections on the machine tool, these connections have different disassembly simplicity. The simplicity of disassembly is a qualitative index, which is generally evaluated by experts before remanufacturing from the aspects of connection structure, connection mode, number of parts and components. In order to simplify and quantify the disassembly simplicity, this paper mainly evaluates the disassembly time, as shown in Formula 1 and 2.

$$\delta = \frac{\sum_{i=1}^{N} c_i \times t_i}{T_d} \quad (i = 1,2,\cdots,N) \tag{1}$$

$$t_d = \begin{cases} 1.00 & \delta \le 1.0 \\ 0.80 & 1.0 < \delta \le 1.2 \\ 0.60 & 1.2 < \delta \le 1.4 \\ 0 & 1.4 < \delta \end{cases} \tag{2}$$

Among them, t_i represents the average disassembly time of the ith connecting parts, c_i represents the number of the ith connecting parts, N represents the number of connecting parts, T_d represents a standard value of disassembly time, δ is an intermediate variable, and t_d represents the disassembly simplicity index value.

In order to make the recycled decommissioned grinders and parts have better reprocessability, the disassembled parts must be cleaned before detection and classification. Different parts need different cleaning methods, and different cleaning methods have different degrees of difficulty. In this paper, the feasibility of cleaning is mainly analyzed from the number of parts and the difficulty of cleaning methods. Based on the investigation of remanufacturing enterprises, it is assumed that the degree of difficulty of various cleaning methods, such as blowing, brushing or baking, chemical detergent spraying and ultrasonic cleaning is 0.20, 0.40, 0.60 and 0.80. The cleaning feasibility index can be calculated by formula 3.

$$t_c = 1 - \frac{\sum_{j=1}^{4} L_j \times \theta_j}{\sum_{j=1}^{4} L_j} \quad (j = 1,2,3,4) \tag{3}$$

Among them, L_j represents the number of parts that are cleaned by the jth washing method, and θ_j represents the difficulty value of the jth washing method.

Performance testing of waste grinder parts is the key link of remanufacturing grinder quality, in order to ensure the quality of remanufacturing grinder, it is necessary to inspect all dismantled parts to ensure that they are not damaged and reusable. Based on the test results of parts, parts can be divided into three categories, that is direct use without reprocessing, usable and unusable after reprocessing. In order to simplify the calculation difficulty, the index value is mainly evaluated by the average detection time and judged by experts. The evaluation result t_i can be divided into four grades (A, B, C, D), and the corresponding index values are (0.95, 0.80, 0.65, 0.50).

Repairing and reprocessing is the key link to restore the waste grinder parts to the new product, it is the most important process of grinder remanufacturing process [19]. Because different users have different working conditions and different usage habits, the quality level of recycled parts varies greatly. Waste grinder parts may have problems in the process of reprocessing and can not continue to use, only by new purchase or new parts to replace. Different parts have different reprocessing processes. Each process has different costs and different success rates. The cost of grinder remanufacturing directly depends on the reuse rate of waste parts. If the waste grinder has good feasibility of parts remanufacturing, it will significantly improve the economy of machine tool remanufacturing. The feasibility index of repairing and reprocessing waste parts can be calculated by formula 4.

$$t_r = \frac{\sum_{k=1}^{N} Q_k \times p_k}{\sum_{k=1}^{N} Q_k} \quad (k = 1,2,\cdots,N) \tag{4}$$

Among them, Q_k represents the number of kth parts after disassembly and cleaning, p_k represents the success rate of repair and reprocessing of kth parts, and N represents the number of parts.

Because the grinder bed and guide rail adopt an integrated structure, the grinder can not be manufactured when the guide rail is seriously worn or deformed and cracks occur, so it can directly recycle resources. Based on the above analysis of each index of technical feasibility and grinder structure form, the evaluation value of technical feasibility can be obtained according to formula 5.

$$T = \alpha\left(\eta_d t_d + \eta_c t_c + \eta_i t_i + \eta_r t_r\right) \tag{5}$$

Among them, η_d, η_c, η_i and η_r represent the weight values of t_d, t_c, t_i and t_r respectively, the weight values can be determined by APH. The value of α is 0 or 1. When the guide of bed can be recycled after repair, α=1. When the guide of bed is seriously worn or corroded, α=0.

3.3. Criteria of economic feasibility

From the aspect of economic feasibility, whether the waste grinder is suitable for remanufacturing is mainly analyzed in terms of making full use of its added value according to the existing technology and technology level to obtain economic benefits. The greater the added value and the lower the remanufacturing cost, the better the economic feasibility and the higher the degree of remanufacturing. In addition, grinder remanufacturing also has significant indirect economic benefits, which can save a large amount of equipment investment and technical training costs of customer enterprises, greatly reduce the cost of purchasing new machine tools of the same performance level, shorten the delivery time by half, and the performance can reach or exceed the original level of new machine tools. This paper mainly judges the economic feasibility of remanufacturing from the cost of grinder remanufacturing, and evaluates the economic benefits of grinder remanufacturing [20].

This paper divides the economic feasibility evaluation index C into two parts, one is to measure the cost of remanufacturing C_1, the other is to measure the cost of new machine tool manufacturing C_2, the specific measurement indicators are as follows.

Grinding machine remanufacturing cost C_1 mainly includes machine tool recovery, disassembly, cleaning, testing, reprocessing and reassembly costs. Each cost mainly includes management cost, material cost, resource cost and human cost. The new manufacturing cost C_2 of grinding machine mainly includes raw material cost, processing cost, assembly cost and quality inspection cost. In the evaluation of the new manufacturing sector, the cost of raw materials includes not only the cost of processing raw materials, but also the purchasing cost of the parts purchased from machine tools.

The evaluation method of economic indicators is different from that of technical indicators. It belongs to quantitative evaluation. Therefore, economic indicators can not be evaluated by analytic hierarchy process. According to Lund Robert in document, if the cost of remanufactured products is 40% to 70% of the price of new products with the same performance, the remanufacturer will benefit. The cost ratio of remanufacturing to new manufacturing is an important parameter to measure the economic feasibility.

Firstly, objective data are obtained by field investigation and evidence collection according to the established indicators, and then the cost C_1 and C_2 under each index of remanufacturing and new manufacturing are obtained by statistical collation of the data.

Secondly, the ratio f of C_1 to C_2 is calculated.

$$f = \frac{C_1}{C_2} \tag{6}$$

Finally, according to the different values of f, the economic feasibility evaluation index C is obtained.

$$C=\begin{cases}1 & f<0.4\\ 1.8-2f & 0.4\le f\le 0.7\\ 0 & 0.7<f\end{cases} \tag{7}$$

When $f < 0.4$, that means the remanufacturing cost is less than 40% of the new manufacturing cost, and the profit of remanufacturing is larger, so the economic feasibility index is 1, which means it is very feasible. When $0.4 < f < 0.7$, the remanufacturing cost is 40% to 70% of the new manufacturing cost of similar grinder products, remanufacturing will be profitable, but the profit margin is inversely proportional to the proportional coefficient. When $0.7 < f$, the remanufacturing cost is more than 70% of the manufacturing cost of the same new machine tool. At this time, the profit margin of remanufacturing is almost zero, so the economic feasibility index is 0, indicating that it is not feasible.

3.4. Criteria of resource environment feasibility

The feasibility of grinder remanufacturing is mainly embodied in three aspects which is resource value, energy value and impact on the environment of the waste grinder. The resource utilization rate, energy saving rate and environmental protection rate are used to evaluate the feasibility of grinder remanufacturing.

The resource utilization rate of remanufacturing resources refers to the parts that are assembled directly by grinding machine. The raw material of remanufacturing grinder comes from waste parts, so its resource utilization rate is reflected in the use of remanufacturing of waste parts. The evaluation index of resource utilization rate of remanufacturing grinder is shown in formula 8.

$$R=\frac{N_u}{N_t}\times\frac{V_u}{V_t} \tag{8}$$

Among them, N_t is the total number of parts for the new grinder, N_u is the number of waste parts for the remanufacturing grinder, V_t is the total value of the new grinder, V_u is the value of the remanufacturing grinder, N_u/N_t is the proportion of the waste parts, V_u/V_t is the utilization value of the waste parts.

The energy saving rate of grinder remanufacturing refers to the amount of energy saved in the process of remanufacturing compared with the energy needed in the new grinder manufacturing. Energy refers to the energy consumed by water, electricity and coal for parts repair and replacement in remanufacturing process. It indirectly participates in the composition of remanufactured products. The evaluation index of remanufacturing energy saving rate is shown in formula 9.

$$P=\frac{1}{n}\sum_{\mathrm{j=1}}^{\mathrm{n}}\frac{V_1-V_2}{V_1} \tag{9}$$

Among them, n is the number of types of energy needed, V_1 is the amount of energy needed for new manufacturing grinders, and V_2 is the amount of energy needed for remanufacturing grinders.

Different from other indicators, it is not easy to quantitatively describe the impact of remanufacturing feasibility of waste grinders on the environment. The purpose of establishing this indicator is to analyze the environmental feasibility of remanufacturing grinders. The evaluation method is to compare the pollutants discharged from remanufacturing process with the national environmental protection standards, and to establish the evaluation index function of environmental protection rate as shown in formula 10.

$$\eta=\begin{cases}1 & x\le q\\ 0 & x>q\end{cases} \tag{10}$$

Among them, x is the discharge of various pollutants in remanufacturing process and q is the discharge of pollutants that meet the national emission standards.

According to the index of resource utilization rate, energy saving rate and environmental protection rate, the comprehensive index E of resource environment evaluation index can be obtained, as shown in formula 11.

$$E=\eta\ (\omega_1 R+\omega_2 P) \tag{11}$$

Among them, ω_1 and ω_2 mean the weight of resource utilization and energy saving rate. According to experience, they are 0.75 and 0.25 respectively.

3.5. Determining weights for individual index by Analytic Hierarchy Process (AHP)

In order to determine the evaluation value of individual index of grinder remanufacturing, the weight value of individual index must be determined first. It is impossible to obtain absolute weights, but only relative weights. AHP is a decision-making method that decomposes the relevant elements into objectives, criteria and schemes, on which qualitative and quantitative analysis can be carried out [21-23].

Because different customers have different requirements for the remanufacturing of used grinders, the weights of individual index are subjective and may vary according to different customer weights. According to the characteristics of AHP method, it is applied to determine the weight of index layer in the feasibility of grinder remanufacturing. The expert platform is composed of customers and the design and management personnel of remanufacturing machine tools. The process of determining the weight of individual index by AHP method is as follows.

Firstly, aiming at the problem of determining the weight of technology feasibility index system, an analytic hierarchy process model is established, as shown in Fig. 6.

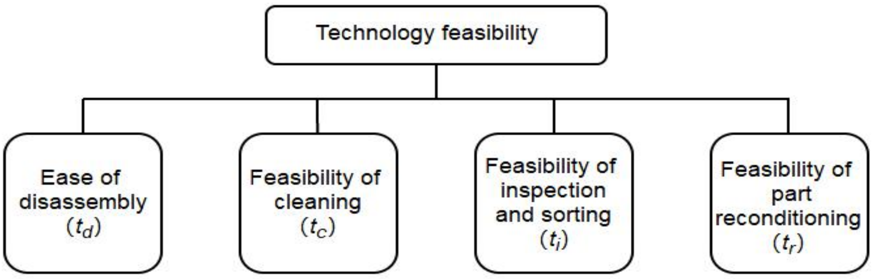

Figure 6: APH model of technology feasibility

Secondly, it establishes the priority judgment scale of technical feasibility indicators, and the value can be obtained by 1 to 9 scale method, as shown in Table 2.

Table 2: Priority judgment scale of technical feasibility elements

Intensity of importance	Definition	notions
1	Equal importance	t_i and t_j contribute equally to the objective
3	Moderate importance	t_i is slightly favored over t_j
5	Obvious importance	Experience and judgment strongly favort_i over t_j
7	Very strong importance	t_i is favored very strongly over t_j
9	Extreme importance	The evidence favoring t_i over t_j is of the highest possible order of affirmation
Intensities of 2, 4, 6, and 8 can be used to express intermediate values		

According to the judgment scale, individual technical feasibility index is compared with each other, and the judgment matrix is established, as shown in Table 3.

Table 3: Judgment matrix of technical feasibility index

	Ease of disassembly (t_d)	Feasibility of cleaning (t_c)	Feasibility of inspection and sorting (t_i)	Feasibility of part reconditioning (t_r)
Ease of disassembly (t_d)	1	3	5	1
Feasibility of cleaning (t_c)	1/3	1	2	1/3
Feasibility of inspection and sorting (t_i)	1/5	1/2	1	1/5
Feasibility of part reconditioning (t_r)	1	3	5	1

Based on AHP, the relative weight of the feasible index of grinder remanufacturing technology can be calculated by formula 12.

$$\omega_i = \frac{\overline{W_i}}{\sum_{i=1}^{n} \overline{W_i}} \quad (i = 1, 2, \cdots, n) \tag{12}$$

Among them, $\overline{W_i} = \sqrt[n]{M_i}$, $M_i = \prod_{j=1}^{n} t_{ij}$.

Finally, the weights of individual technical feasibility index are determined by AHP, and the results are shown in formula 13.

$$W = \{t_d, t_c, t_i, t_r\} = \{0.3937,\ 0.1374,\ 0.0752,\ 0.3937\} \tag{13}$$

4. Comprehensive benefit evaluation for grinder remanufacturing

4.1. Contents of comprehensive benefit evaluation

The comprehensive benefit evaluation of grinder remanufacturing is a complex system with fuzziness. The benefit of grinder remanufacturing is hierarchical. The technical benefit of remanufacturing can be divided into product quality, timeliness of remanufacturing and upgrading of product technology. Because the comprehensive benefit evaluation involves many factors, but lacks data, many factors are in the state of fuzzy qualitative analysis, so the comprehensive benefit evaluation of grinder remanufacturing can adopt the fuzzy APH [24].

The evaluation of grinder remanufacturing benefits should be based on technical benefits, including technical benefits, economic benefits, environmental and resource benefits. Technical benefit refers to product energy efficiency, remanufacturing product quality and remanufacturing time after remanufacturing activities, including comprehensive technical upgrading of grinders after remanufacturing. Economic benefit refers to the benefit of remanufacturing activities in product life-cycle cost management. Environmental benefit refers to the resource saving and environmental protection benefits of remanufacturing activities.

The evaluation system of remanufacturing benefits studied in this paper is divided into two levels. The first level evaluation index is technical, economic and environmental benefits of grinder remanufacturing, which constitutes the first level evaluation factor set *U*.

$$U = (u_1, u_2, u_3) \tag{14}$$

Based on the analysis of grinder remanufacturing practice, the technical, economic and environmental benefits are subdivided into two levels to form a set of secondary evaluation factors. The comprehensive benefit evaluation system of grinder remanufacturing is shown in Table 4.

According to the experience of grinder remanufacturing and the actual needs, the commentary set *V* is determined.

$$V = (v_1, v_2, v_3, v_4) \tag{15}$$

The comment set V is divided into four grades, v_1 is excellent, v_2 is good, v_3 is general, and v_4 is poor.

Table 4: Comprehensive benefit evaluation system of grinder remanufacturing

Target (U)	First level index (u_i)	Second level index (u_{ij})
Comprehensive benefit of remanufacturing grinder	Technical benefit	Modularization level
		Standardization level
		Ease of reassembly
		Machine upgrading
		Technical maturity
	Economic benefit	Remanufacturing cost
		Remanufacturing profit
		Maintenance cost
	Environmental benefit	Material reuse rate
		Energy conservation rate

4.2. Comprehensive benefit evaluation system

The evaluation of comprehensive benefit weight set of grinder remanufacturing can be determined by combining AHP with Expert Investigation Method (Delphi), which is similar to the determination of technical feasibility index weight in Chapter 3.5. By comparing the importance of each level of indicators in the comprehensive benefit evaluation model, the judgment matrix A_i of each level of evaluation indicators is obtained, and then the maximum eigenvalue λ_i and corresponding eigenvector P_i of each judgment matrix are obtained, the consistency test of λ_i is carried out. The CI and CR are shown in the formula 16, RI is the average random consistency index, the values are shown in Table 5.

$$\begin{cases} CI = \dfrac{\lambda_i - n}{n - 1} \\ CR = \dfrac{C}{RI} \end{cases} \tag{16}$$

Table 5: RI values of order 1 to 7 matrices

Order number	1	2	3	4	5	6	7
RI	0.00	0.00	0.58	0.90	1.12	1.24	1.32

When CR is less than 0.1, the consistency of the judgment matrix is acceptable. Otherwise, Delphi method should be used again to compare the importance and construct the judgment matrix until CR is less than 0.1. Finally, the weight set of the first-level index and the weight set of the second-level index are obtained.

For each factor u_i, it evaluates each aspect of comment set V separately, forming a single factor evaluation fuzzy subset. Expert evaluation method can be used to determine the membership degree of each index. Several experts in the field of remanufacturing can be invited to form an evaluation expert group, and their evaluation can be indicated by scoring. r_{ij} is the membership degree of any index u_i in the index set U to the elements in the comment set V, their relations are as follows.

$$r_{ij} = \frac{c_{ij}}{\sum_{j=1}^{m} c_{ij}}, i = 1,2,\cdots,n \tag{17}$$

Among them, $\sum_{j=1}^{m} c_{ij}$ represents the number of expert groups, c_{ij} means the number of votes in favour of factor u_i and evaluation v_j, and the single factor membership matrix R_i can be obtained.

$$R_i = \begin{pmatrix} r_{11} & \cdots & r_{1m} \\ \vdots & \cdots & \vdots \\ r_{n1} & \cdots & r_{nm} \end{pmatrix} \tag{18}$$

The weight vector of the first grade index is W_i by single factor fuzzy evaluation, and the comprehensive benefit evaluation result B_i of the first grade grinder remanufacturing can be obtained by using the weighted sum algorithm.

$$B_i = W_i R_i \tag{19}$$

The comprehensive benefit evaluation of fuzzy remanufacturing was calculated, the three sets of B_i are taken as a subset of the first-level comprehensive evaluation, and the fuzzy comprehensive evaluation matrix $R=(B_1, B_2, B_3)^T$ is constructed. Then the mathematical model of fuzzy comprehensive benefit evaluation for grinder remanufacturing is as follows.

$$B = WR \tag{20}$$

The percentile system is used to assign the four grades of the comment set V. Among them, v_1 is excellent and the assignment score is 90 to 100, v_2 is good and the assignment score is 80 to 89, v_3 is general and the assignment score is 65 to 79, v_4 is poor and the assignment score is 50 to 64. Quantitative value of fuzzy comprehensive benefit evaluation for remanufacturing was obtained, and the performance level of the remanufactured grinder was determined according to the remanufacturing comment set V.

5. Case study of remanufacturing grinder

5.1. Feasibility analysis of grinder remanufacturing

At present, there are more than 9 million machine tools in China, but the overall level of domestic machine tools is relatively backward. According to statistics, more than 60% of machine tools have been in service for more than 10 years. These machine tools may face scrap, idle or functional elimination in the next 5 to 10 years, thus forming a considerable potential resource for machine tool remanufacturing.

In an engine parts manufacturer, Three M1332B cylindrical grinders made by Shanghai Machine Tool Works Company have been in service for more than 15 years and are facing scrap and technology elimination, The retired M1332B cylindrical grinder is shown in the Fig. 7. The company wants to meet the processing needs and save labor costs by purchasing three CNC cylindrical grinders of the same specifications. However, due to the high price of CNC cylindrical grinders, enterprises can not afford to pay for three new grinders. Because of the high residual value of waste cylindrical grinders, enterprises choose to remanufacture grinders and upgrade them numerically to meet production needs. The cylindrical grinder is the main equipment of the enterprise. The original machine tool uses relay to control logically. The worktable is driven by hydraulic cylinder. The control technology is backward and the failure rate of the machine tool is high, but the mechanical accuracy is high. According to the requirements of enterprises, the grinder after remanufacturing is controlled by CNC system, and the feed mode of worktable is ball screw and servo motor. One worker can operate three grinders at the same time. Following is the detailed process of feasibility analysis for remanufacturing of cylindrical grinder.

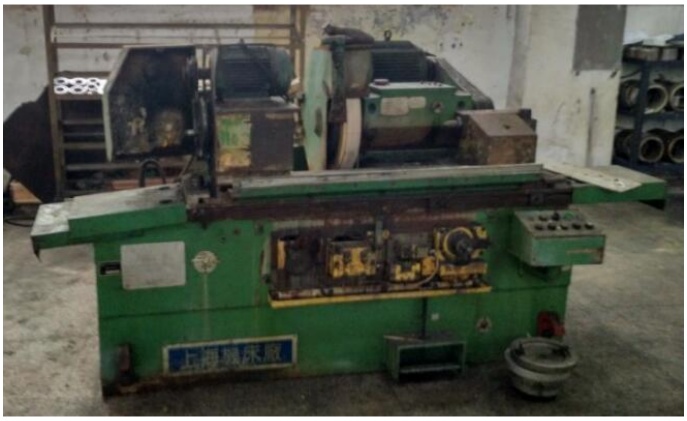

Figure 7: The retired M1332B cylindrical grinding machine.

Firstly, the technical feasibility of remanufacturing of waste M1332B cylindrical grinder is analyzed. According to the evaluation index method proposed in this paper, the technical feasibility evaluation results can be obtained, as shown in Table 6.

Table 6: Technical feasibility evaluation results

Index	Notions	Evaluation value
Ease of disassembly	t_d	0.85
Feasibility of cleaning	t_c	0.75
Feasibility of inspection and sorting	t_i	0.82
Feasibility of part reconditioning	t_r	0.83
Wear condition of guide rail	α	1.00

The remanufacturing technical feasibility of M1332B cylindrical grinder can be calculated by formula 5. The evaluation results are as follows.

$$\begin{aligned} T &= \alpha\left(\eta_d t_d + \eta_c t_c + \eta_i t_i + \eta_r t_r\right) \\ &= 1 \times \left(0.3937 \times 0.85 + 0.1374 \times 0.75 + 0.0752 \times 0.82 + 0.3937 \times 0.83\right) \\ &= 0.826 \end{aligned}$$

According to the evaluation results, the remanufacturing technical feasibility of cylindrical grinder grinder is better, and the remanufacturing feasibility evaluation process of grinder can enter the next step.

The second step is to evaluate the remanufacturing economic feasibility of M1332B cylindrical grinder and calculate the remanufacturing cost to ensure that the remanufacturer is profitable. According to customer demand, the price of purchasing MKA1332 simple CNC cylindrical grinder with the same performance level needs RMB 320,000, while three such grinders need RMB 960,000. The cost of upgrading a retired M1332B cylindrical grinder to MKA1332 CNC cylindrical grinder is about RMB 145,000. The remanufacturing economic feasibility of grinders can be calculated by formula 6. The evaluation results are as follows.

$$f = \frac{C_1}{C_2} = \frac{145000}{320000} = 0.453$$

According to the value of f, the economic feasibility evaluation index C can be obtained.

$$C = 1.8 - 2f = 1.8 - 2 \times 0.453 = 0.894$$

The economic feasibility evaluation results of remanufacturing show that the grinder has good economic feasibility, and the remanufacturer can obtain a larger profit, with remarkable economic benefits. The feasibility evaluation process of remanufacturing can enter the next step.

Finally, the feasibility of resources and environment for remanufacturing of M1332B cylindrical grinder is analyzed from the aspects of resource saving, energy saving and environmental protection. According to the evaluation method proposed in this paper, each index of resource and environment feasibility is quantified, and the result of resource and environment feasibility evaluation can be obtained, as shown in table 7.

Table 7: Resources environment evaluation results

Index	Notions	Evaluation value
Resource utilization rate	R	0.834
Energy conservation rate	P	0.917
Environmental protection rate	η	1.00

Subsequently, the remanufacturing resources environmental feasibility of M1332B cylindrical grinder can be calculated by formula 11, and the evaluation results are as follows.

$$\begin{aligned} E &= \eta\ (\omega_1 R + \omega_2 P) \\ &= 1 \times (0.75 \times 0.834 + 0.25 \times 0.917) \\ &= 0.855 \end{aligned}$$

From the results of resource environment feasibility assessment, it can be seen that the remanufacturing of waste M1332B cylindrical grinder has better resource conservation and environmental friendliness. The grinder is suitable for remanufacturing and can maximize the reuse of waste resources.

5.2. Process analysis of grinder remanufacturing

M1332B cylindrical grinder mainly consists of bed, worktable, grinding wheel rack, head rack, tail rack, hydraulic system, cooling system, protective cover, electrical system and so on. After disassembly, cleaning, testing and classification, the mechanical parts of the external grinder are repaired and reprocessed, the CNC system, servo motor and ball screw pairs are added, the electrical system and cooling system are replaced and upgraded, and parts are replaced to restore the accuracy of the machine tool to meet the factory standards to meet customer requirements.

After disassembly and cleaning, the loss of parts and precision of cylindrical grinder are analyzed, and the remanufacturing process plan is worked out. The remanufacturing process method and process of M1332B cylindrical grinder are as follows.

The repairing of grinder bed guide rail will cause different degree of wear and tear during the use of cylindrical grinder, which will affect the accuracy of machine tool processing. The guide rail of the bed casting can be repaired with casting adhesives when the wear is not serious. Then the guide rail grinder is used to grind the guide rail to the standard size. Finally, manual scraping of the bed guide rail is carried out. The purpose of scraping is to increase the actual contact area of the guide rail surface when working, and to store a small amount of lubricant in the scraping pit to reduce the friction of the moving contact surface and prolong the service life of the guide rail. The grinder integrated guideway is reconditioned as shown in Fig. 8.

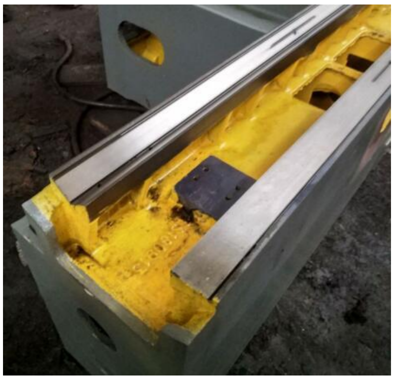

Figure 8: Reconditioned grinding machine integrated guideway.

The grinding wheel frame spindle system is the core component of the cylindrical grinder. The remanufacturing results of the grinding wheel frame will directly affect the accuracy of the grinder. The remanufacturing of the grinding wheel frame includes the repair of the spindle and the replacement of the hydrodynamic and hydrostatic bearings. The surface defect of grinding wheel rack spindle is repaired by coating method, and the roundness error of the spindle is less than 0.002 mm by grinding, the surface roughness can meet the requirements of Ra 0.2um. The gap between hydrodynamic bearing and spindle should meet the requirements of 0.015 mm to 0.025 mm. After reconditioning, the grinding wheel frame spindle system is shown in Fig. 9.

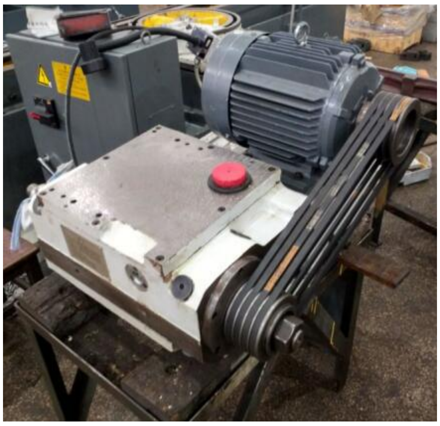

Figure 9: Reconditioned grinding wheel frame spindle system.

Trapezoidal screw is used in the original drive mode of cylindrical grinder, which has been worn seriously. In order to ensure the motion accuracy and improve the motion flexibility, ball screw should be replaced. The improvement of grinder feed system is mainly to shorten the feed drive chain and drive the lead screw directly by servo motor, which can greatly reduce the error transmission between all levels of the drive chain. At the same time, servo motor drive can form semi-closed-loop control to improve machine tool processing accuracy. The feed system of cylindrical grinder before and after reconditioning are shown in Fig. 10 and Fig. 11.

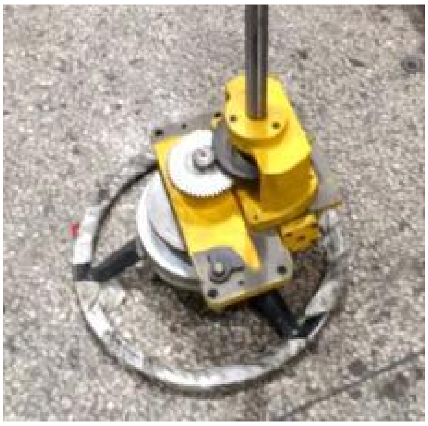

Figure 10: Grinding machine feed system before reconditioning.

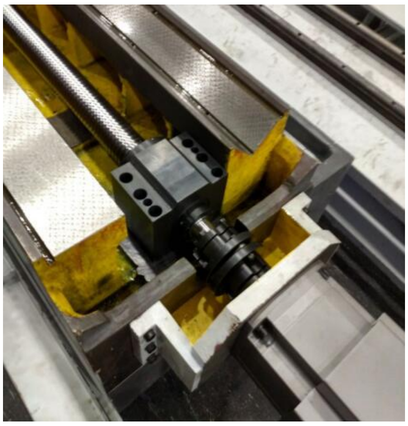
Figure 11: Reconditioned grinding machine feed system.

Retired M1332B cylindrical grinder is a manual grinder, which has no CNC system and has low control performance. It is difficult to meet the requirements of multi-batch and high efficiency processing parts. According to the principle of grinder remanufacturing combined with customer requirements, the electrical system remanufacturing scheme of cylindrical grinder is to install KT630G1 grinder CNC system, driver and control panel of Shanghai Capital NC Company, which can simultaneously realize rapid response and accurate control of output of each axis. Because the electrical control system of M1332B cylindrical grinder is completely different from that of CNC cylindrical grinder, and the electrical components of waste cylindrical grinder have been seriously aging, the original electrical control system of cylindrical grinder needs to be replaced, and the intensive power control box should be redesigned and manufactured. The intensive power control box and CNC system of M1332B cylindrical grinder before and after reconditioning are shown in Fig. 12 and Fig. 13.

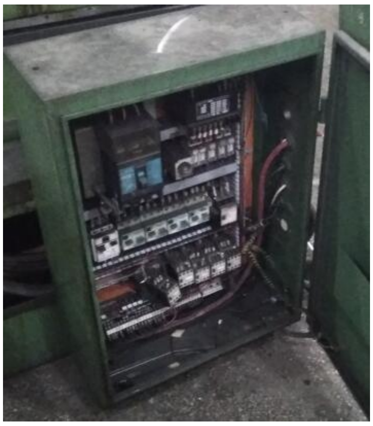
Figure 12: Grinder intensive power control box before reconditioning.

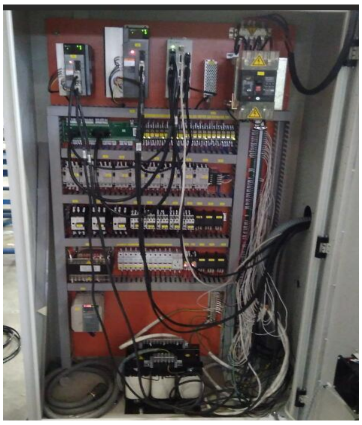
Figure 13: Reconditioned grinder intensive power control box and CNC system.

After repairing and reprocessing the parts, adjust the gap of the parts, scrape the joints of the parts, reassemble

according to the assembly process, and restore the accuracy of the machine tool. Components assembled on remanufactured machine tools should meet the quality requirements. The remanufactured cylindrical grinder M1332B shall be inspected and checked according to the factory standard of the new machine tool. The results of comparison between the mechanical geometric accuracy of remanufactured cylindrical grinder and the factory standard are shown in Table 8.

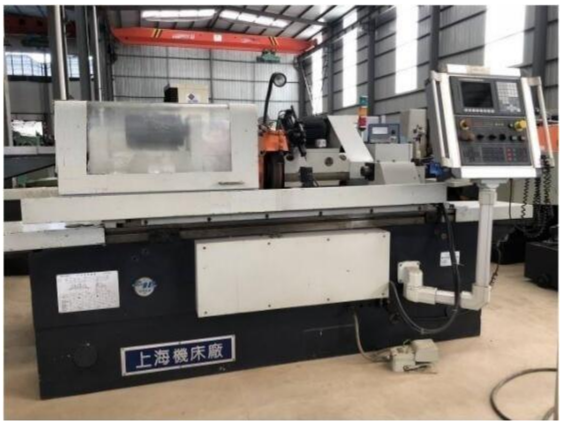

Figure 14: The remanufactured M1332B cylindrical grinding machine.

Table 8: Acceptance results of remanufactured grinder geometric accuracy

Number	Inspection items	Factory standard (mm)	Remanufactured grinder (mm）
G1	Straightness of workbench moving (Z-axis) in Z-X horizontal plane	0.015	0.012
G2	Straightness of grinding wheel frame moving (X-axis) in Z-X horizontal plane	0.02	0.017
G3	Verticality of grinding wheel rack movement (X-axis) to table movement (Z-axis)	0.015	0.013
G4	Parallelism of headframe spindle rotary axis to workbench movement (Z-axis)	0.008	0.008
G5	Parallelism of taper hole axis of tailstock sleeve to workbench movement (Z-axis)	0.008	0.006
G6	Radial runout of grinding wheel frame spindle	0.005	0.005
G7	Parallelism of grinding wheel spindle axis to table movement (Z-axis)	0.015	0.012
G8	Equal height of headframe spindle axis and grinding wheel spindle axis to reference plane (plane constituted by X-axis and Z-axis Moving)	0.30	0.24

Table 8 shows that the geometric accuracy of remanufactured cylindrical grinder meets the factory standard completely, and some indexes are even better. In addition, CNC system is added to remanufacturing grinder to realize CNC operation. According to customer needs, intelligent and automation modules can be added in the follow-up. The state of remanufactured M1332B cylindrical grinder is shown in Fig. 14.

Casting parts such as bed, workbench, grinding wheel frame, headframe and tailstock of waste M1332B cylindrical grinder and other parts with higher added value have been reused. The utilization rate of resource recycling is over 80% by weight, and the mechanical part of the machine tool has durability and stable performance. Especially for the bed

castings, the longer the aging time, the better the performance, the more stable and reliable the machine tool performance after remanufacturing.

Acceptance check of the working accuracy of the remanufactured cylindrical grinder M1332B. A 32x315mm alloy steel specimen was used. The rotational speed of the specimen was 80 to 130 r/min, and the working table speed was 0.5 to 1.5m/min. As shown in Table 9, the working accuracy of remanufactured cylindrical grinder is compared with the factory standard.

Table 9: Working accuracy of remanufacturing grinder

Number	Inspection items	Factory standard	After remanufacturing
P1	Roundness (um)	1.5	1.1
P2	Uniformity of longitudinal Section diameter (um)	3	2.3
P3	Surface roughness (Ra um)	0.32	0.16

According to the acceptance check of the working accuracy index of remanufactured M1332B cylindrical grinder, it is obvious that the remanufactured machine tool fully meets the factory standard of the new machine tool, and some of the indexes are better. The cost of remanufactured cylindrical grinder is over 50% less than that of newly purchased grinder, and the performance of machine tool is better than that of new machine tool.

5.3. Comprehensive benefit analysis of grinder remanufacturing

The comprehensive benefit evaluation of remanufactured M1332B cylindrical grinder is carried out, the evaluation index factors are set up in Table 4. With using the scaling method of scale 1 to 9 in Table 2, the judgment matrix of the first-level index relative to the target level and the second-level index relative to the first-level index is determined. The matrices are shown in Table 10, 11 and 12.

Table 10: First level index judgment matrix A

	Technical benefit (u_1)	Economic benefit (u_2)	Environmental benefit (u_3)
Technical benefit (u_1)	1	3	8
Economic benefit (u_2)	1/3	1	3
Environmental benefit (u_3)	1/8	1/3	1

Table 11: Second level index judgment matrix A_1 of technique

	Modularization level (u_{11})	Standardization level (u_{12})	Ease of reassembly (u_{13})	Machine upgrading (u_{14})	Technical maturity (u_{15})
Modularization level (u_{11})	1	2	5	2	4
Standardization level (u_{12})	1/2	1	3	1	2
Ease of reassembly (u_{13})	1/5	1/3	1	1/2	1/2
Machine upgrading (u_{14})	1/2	1	2	1	1/2
Technical maturity (u_{15})	1/4	1/2	2	1/2	1

Table 12: Second level index judgment matrix A_2 of economics

	Remanufacturing cost (u_{21})	Remanufacturing profit (u_{22})	Maintenance cost (u_{23})
Remanufacturing cost (u_{21})	1	2	4
Remanufacturing profit (u_{22})	1/2	1	2
Maintenance cost (u_{23})	1/4	1/2	1

The weight of individual secondary index and primary index for the comprehensive benefit evaluation of grinder remanufacturing is calculated, and the consistency is verified. For the first-level index judgment matrix A, the normalized results are as follows.

W=(0.6817, 0.2364, 0.0819), Its maximum eigenvalue λ_i=3.0015, CI=0.0008, RI=0.58, CR=0.0014.

Similarly, the calculation and consistency test of the second-level weight indexes for the comprehensive benefit evaluation of grinder remanufacturing are as follows.

W_1=(0.4073, 0.2112, 0.0748, 0.1948, 0.1119), Its maximum eigenvalue λ_i=5.0415, CI=0.0104, RI=1.12, CR=0.0093.

W_2=(0.5714, 0.2857, 0.1429), Its maximum eigenvalue λ_i=3.0000, CI=0.0000, RI=0.58, CR=0.0000.

According to the experience, there are only two factors in environmental benefit index u_3, the weight of environmental benefit index can be obtained as W_3=(0.7500, 0.2500).

Finally, according to the results of cylindrical grinder remanufacturing, 20 experts who have been engaged in machine tool remanufacturing field for a long time are invited to form an evaluation group to evaluate the remanufacturing cylindrical grinder comprehensively by voting. The specific evaluation results are shown in Table 13.

Table 13: Expert evaluation results

Index attribute	Evaluation results (unit numbers)			
	Excellent evaluation	Good evaluation	General evaluation	Poor evaluation
Modularization level (u_{11})	9	8	2	1
Standardization level (u_{12})	5	11	3	1
Ease of reassembly (u_{13})	6	9	3	2
Machine upgrading (u_{14})	12	6	2	0
Technical maturity (u_{15})	14	4	2	0
Remanufacturing cost (u_{21})	12	6	2	0
Remanufacturing profit (u_{22})	7	6	5	2
Maintenance cost (u_{23})	8	5	4	3
Material reuse rate (u_{31})	12	5	3	0
Energy conservation rate (u_{32})	8	9	2	1

From Table 13, the membership degree of each influencing factor of the comprehensive benefit evaluation index of

remanufacturing can be calculated, and the single factor membership degree matrix can be obtained. Taking technical benefit factors as an example, the technical membership matrix is obtained as follows.

$$R_1 = \begin{pmatrix} 0.45 & 0.40 & 0.10 & 0.05 \\ 0.25 & 0.55 & 0.15 & 0.05 \\ 0.30 & 0.45 & 0.15 & 0.10 \\ 0.60 & 0.30 & 0.10 & 0 \\ 0.70 & 0.20 & 0.10 & 0 \end{pmatrix}$$

According to formula 19.

$$B_1 = W_1 R_1 = \begin{pmatrix} 0.4073 \\ 0.2112 \\ 0.0748 \\ 0.1948 \\ 0.1119 \end{pmatrix}^{\mathrm{T}} \begin{pmatrix} 0.45 & 0.40 & 0.10 & 0.05 \\ 0.25 & 0.55 & 0.15 & 0.05 \\ 0.30 & 0.45 & 0.15 & 0.10 \\ 0.60 & 0.30 & 0.10 & 0 \\ 0.70 & 0.20 & 0.10 & 0 \end{pmatrix} = (0.4537, 0.3936, 0.1143, 0.0384)$$

Similar problem can be explained.

$$B_2 = W_2 R_2 = (0.5, 0.2929, 0.1571, 0.05)$$

$$B_3 = W_3 R_3 = (0.55, 0.3, 0.1375, 0.0125)$$

According to formula 20 and the fuzzy comprehensive evaluation matrix $R=(B_1 \quad B_2 \quad B_3)$, the comprehensive benefit evaluation results of grinder remanufacturing are as follows.

$$B = WR = \begin{pmatrix} 0.6817 \\ 0.2363 \\ 0.0819 \end{pmatrix}^{\mathrm{T}} \begin{pmatrix} 0.4537 & 0.3936 & 0.1143 & 0.0384 \\ 0.5000 & 0.2929 & 0.1571 & 0.0500 \\ 0.5500 & 0.3000 & 0.1375 & 0.0125 \end{pmatrix} = (0.4725, 0.3621, 0.1263, 0.0390)$$

In order to transform the weight distribution of the four grades in the final evaluation set into a total score, the evaluation grades are quantified and assigned to the four grades by percentage system. Among them, the excellent value is 95 points, the good value is 85 points, the general value is 72 points and the poor value is 57 points. Then the calculation result of the comprehensive benefit evaluation value Q for M1332B cylindrical grinder remanufacturing is as follows.

$$\begin{aligned} Q &= 0.4725v_1 + 0.3621v_2 + 0.1263v_3 + 0.039v_4 \\ &= 0.4725 \times 95 + 0.3621 \times 85 + 0.1263 \times 72 + 0.039 \times 57 \\ &= 86.9826 \end{aligned}$$

According to the comprehensive evaluation of remanufacturing benefit set V, it can be seen that the comprehensive benefit of remanufacturing M1332B cylindrical grinder is at a good level, close to the excellent level. The remanufacturing grinder has reached the expected level demanded by customers, and the waste resources and materials have been optimized.

6. Conclusion

This paper describes the concept of grinder remanufacturing. A comprehensive method for evaluating the feasibility of grinder remanufacturing is put forward from four aspects which is grinder structure, technical feasibility, economic feasibility and resource environment feasibility, a feasibility evaluation system for grinder remanufacturing is established. According to the analysis of grinder remanufacturing process, APH method is used to determine the weight of each element in the evaluation system. Finally, the comprehensive benefit of grinder remanufacturing is determined

by combining AHP with Delphi, and the grade level of remanufacturing grinder is determined by the comprehensive benefit evaluation value Q of grinder remanufacturing.

The research results of this paper prove the feasibility of remanufacturing grinder and the practicability of comprehensive benefit evaluation. Although the establishment of the evaluation system for grinder remanufacturing has achieved good results, the accuracy of the parameters of the evaluation system is limited in practical application. In order to improve the authenticity of the evaluation system, it is necessary to further study the remanufacturing process of grinders, and to analyze the remanufacturing cases of different types of grinders for the sake of obtaining more remanufacturing experience data. In addition, the evaluation system of remanufacturing of retired grinders can be extended to the remanufacturing systems of other mechanical products, and remanufacturing has become an important part of the mechanical engineering industry. Remanufacturing can not only prolong the life cycle of mechanical products and reduce the cost, but also embodies the realization of energy saving, emission reduction and green development. It will become an important direction for the future development of mechanical engineering.

Conflict of Interests

The authors declare that there is no conflict of interests regarding the publication of this paper.

Acknowledgments

This research work was supported by Shanghai Science and Technology Development Foundation (CN) (NO Z16030384).

Reference

[1] RT Lund,"Remanufacturing, United States Experience and Implications for Developing Nations," Center for Policy Alternatives, Massachusetts Institute of Technology, 1983.

[2] S Ullah, I Muhammad, TJ Ko,"Optimal strategy to deal with decision making problems in machine tools remanufacturing," International Journal of Precision Engineering and Manufacturing-Green Technology, vol.3, no.1, pp.19-26, 2016.

[3] S Bernard,"Remanufacturing," Journal of Environmental Economics and Management, vol.62, no.3, pp.337-351, 2011.

[4] W Liu, B Zhang, Y Li, Y He, and H Zhang,"An environmentally friendly approach for contaminants removal using supercritical CO_2 for remanufacturing industry," Applied Surface Science, vol.292, pp.142-148, 2014.

[5] F Zhou, Z Jiang, H Zhang, and Y Wang,"A Case-Based Reasoning Method for Remanufacturing Process Planning," Discrete Dynamics in Nature and Society, vol.2014, 2014.

[6] RB Aronson,"Remanufactured Machine Tools," Manufacturing Engineering, vol.131, No.6, pp.75-80, 2003.

[7] Du Y., Li C.,"Implementing energy-saving and environmental-benign paradigm: machine tool remanufacturing by OEMs in China," Journal of Cleaner Production, vol.66, pp.272-279, 2014.

[8] Bin-Shi Xu,"Progress of remanufacturing engineering and future technology expectation," Advances in Manufacturing, vol.1, no.1, pp.8-12, 2013.

[9] S. S. Yang, N. Nasr, S. K. Ong, A. Y. C. Nee,"A holistic decision support tool for remanufacturing: end-of-life (EOL) strategy planning," Advances in Manufacturing, vol.4, no.3, pp.189-201, 2016.

[10] A Rashid, FMA Asif, P Krajnik,"Resource conservative manufacturing: an essential change in business and technology paradigm for sustainable manufacturing," Journal of Cleaner Production, vol.57, no.15, pp.166-177, 2013.

[11] Z Jiang, S Zhu, H Zhang, Y Wang,"Optimal Acquisition and Inventory Control for a Remanufacturing System," Discrete Dynamics in Nature and Society, vol.2013, 2013.

[12] D Pigosso, ET Zanette, AG Filho, AR Ometto,"Ecodesign methods focused on remanufacturing," Journal of Cleaner Production, vol.18, no.1, pp. 21-31, 2010.

[13] S Pokharel, Y Liang,"A model to evaluate acquisition price and quantity of used products for remanufacturing," International Journal of Production Economics, vol.138, no.1, pp.170-176, 2012.

[14] Matsumoto M, Naito K,"A study on remanufacturing businesses in Japan," 2009 IEEE International Symposium on Sustainable Systems and Technology, IEEE Computer Society Washington, DC, USA, pp.1-6, 2009.

[15] R Subramoniam, D Huisingh, RB Chinnam,"Remanufacturing for the automotive aftermarket-strategic factors: literature review and future research needs," Journal of Cleaner Production, vol.17, no.13, pp.1163-1174, 2009.

[16] Li C.B., Liu F., Cao H.J., Wang Q.L.,"A stochastic dynamic programming based model for uncertain production planning of remanufacturing system," International Journal of Production Research, vol.47, no.13, pp.3657-3668, 2009.
[17] P Zwolinski, MA Lopez-Ontiveros, D Brissaud,"Integrated design of remanufacturable products based on product profiles," Journal of Cleaner Production, vol.14, no.15, pp.1333-1345, 2006.
[18] Guide, V. D. R.,"Production planning and control for remanufacturing: industry practice and research needs," Journal of Operations Managemen, vol.18, no.4, pp.467-478, 2000.
[19] Ferguson, M., Guide, V. D., Koca, E., Souza, G.C.,"The value of quality grading in remanufacturing," Production and Operations Management Society, vol.18, no.3, pp.300-314, 2009.
[20] MR Johnson, Evaluating Remanufacturing and Demanufacturing for Extended Producer Responsibility & Sustainable Product Management [Ph.D. thesis], University of Windsor, Canada, 2002.
[21] TL Saaty,"Decision making with the analytic hierarchy process," International Journal of Service Sciences, vol.1, no.1, pp.83-98, 2008.
[22] A Shapira, M Simcha,"AHP-based weighting of factors affecting safety on construction sites with tower cranes," Journal of Construction Engineering and Management, vol.135, no.4, pp.307-318, 2009.
[23] P Korhonen, R Voutilainen,"Finding the most preferred alliance structure between banks and insurance companies," European Journal of Operational Research, vol.175, no.2, pp.1285-1299, 2006.
[24] Zeki Ayağ, R. G. Özdemir,"A Fuzzy AHP Approach to Evaluating Machine Tool Alternatives," Journal of Intelligent Manufacturing, vol.17, no.2, pp.179-190, 2006.
