# Supplementary material for: The remanufacturing evaluation for feasibility and comprehensive benefit of retired grinding machine
Source: PLoS One. 2020 Jun 18;15(6):e0234603. doi: 10.1371/journal.pone.0234603 (PMC7302915; doi:10.1371/journal.pone.0234603)
Supplement: Attachment — Submitted filename: Reviewer-PONE-19-28523.docx [file pone.0234603.s001.docx]

***Dear editor in chief:***

This paper is good and interesting, written in suitable way, this paper described the concept of grinder remanufacturing. A comprehensive method for evaluating the feasibility of grinder remanufacturing is put forward from four aspects which is grinder structure, technical feasibility, economic feasibility and resource environment feasibility.

But I am not sure if the paper met the minimum requirement of plos one journal, as you know Q1 should has novelty or high contribution. Anyway, I have been written some comments that will enhanced the quality of paper to be more suitable.

1.	Abstract needs some improvements by highlight the contributions and main findings.

2.	The authors developed a strong methodological approach, but a theoretical background strengthening is recommended by adding more modern studies.

3.	please standardize the writing of “fig or figure”.

4.	The conclusion drawn from the study are acceptable but some of the conclusions are not mentioned. The conclusion should extract from result and discussion. Conclusions need improvements, could you highlight the main finding?

5.	The contribution is not much clear, what is the difference with other researchers, could you provide comparison with others? Could you please highlight more the contribution of your work?

6.	I highly recommend the authors also to focus their revised manuscript on the issues of: "Technology maturity" and "Economics" (or "Pricing") upon technologies discussed. This extra information could be extracted from either the existing 24 citations, or those that the authors will decide to include in their revised manuscript. This extra information should be gathered and given in quantitative or qualitative format, either in the "4. Discussion" or in the "5. Conclusion" sections.

7.	Based on your experience, could you please add some future directions in this article in the conclusion part.

8.	Please check the reference formatting writing according to Plos One journal standard.

Perhaps, the paper doesn’t present high level of new contribution or novelty, but I think the paper is could be accepted after making major corrections.
